# Animal Chemistry

**Published:** 1836-04

**Authors:** 


### ANIMAL CHEMISTRY.

*Researches on the Blood.* By L. Gmelin, F. Tiedemann, and E. Mitscherlich.

[These important researches embrace three subjects of enquiry, viz. 1. the carbonic acid of the blood; 2. the state of the blood after extirpation of the kidneys; and 3. whether urea or sugar of milk can be detected in healthy blood. In presenting them to our readers, we shall content ourselves with giving the substance of the memoir, without adhering to the exact words of the distinguished authors; and we shall introduce, as we go along, such remarks as suggest themselves to us.]

### § 1. *The Carbonic Acid of the Blood.*

Though the question whether carbonic acid exists in the blood, either free or combined with a base, is one of considerable moment, especially as regards the theory of respiration, the experiments undertaken by different chemists, with a view of determining it, have afforded very contradictory results. In the present experiments one source of fallacy has been carefully guarded against, namely, the access of atmospheric air to the blood submitted to examination. A small metallic tube, with a stopcock, was inserted into the femoral artery, and a similar one into the femoral vein, of a living dog; to each of the tubes an elastic gum catheter was adapted, along which the blood was permitted to flow, till the contained air was wholly expelled; the end of the catheter was then brought under a glass jar, three and a half inches high, and one inch wide, previously filled with and inverted over mercury, and the blood was allowed to rise till it filled the upper half of the vessel, which was then conveyed under the receiver of an air pump.

At first, no air bubbles were emitted under the action of the pump from either kind of blood, nor until the exhaustion reached twenty-seven or twenty-eight inches of the barometer; at which time a quantity of aeriform fluid was evolved from both, so as to depress the mercury in the jar about an inch. On re-admitting the air, however, the evolved fluid suddenly disappeared, long before the atmospheric pressure was wholly restored, from whence the experimenters inferred, that it was merely watery vapour, generated in a Torricellian vacuum, and not a permanent gas. From this experiment, accordingly, they conclude that neither arterial nor venous blood contains free carbonic acid.

The experiment just related undoubtedly proves that no carbonic acid or other gas is emitted from blood on removing atmospheric pressure, a result which agrees with that previously obtained by Dr. Darwin and by Dr. John Davy, and is further confirmed by experiments made almost simultaneously with the above, but independently, by Stromeyer, and by Dr. J. Müller, of Berlin. But it is contended by Dr. Stevens, that the non-evolution of carbonic acid from the blood under the air pump does not amount to a proof of its non-existence in that fluid, since serum of blood purposely impregnated with carbonic acid cannot be made to yield it again by the mere removal of the atmospheric pressure: moreover, to show positively that free carbonic acid exists in the blood, he adduces an experiment, in which he obtained carbonic acid from venous blood on agitating it with hydrogen, and exposing it for an hour to the contact of that gas.* Without pretending here to discuss the question, we may be permitted to remark, first, that carbonic acid purposely added to blood may be fixed and retained in it by the soda which the blood contains, and secondly, that Dr. J. Müller could obtain no appreciable quantity of carbonic acid from blood on agitating it *for a long time* with hydrogen.† It seems therefore desirable that Dr. Stevens should repeat his experiment, with a view of determining the *amount* of carbonic acid extracted in this way from a given quantity of blood.

The authors mention as a fact worthy of notice, that coagulation took place as usual, though the access of air was prevented, and that the characteristic difference of colour of the two kinds of blood was obvious during the whole experiment, showing that the brighter colour of arterial blood does not depend on a difference in its mode of coagulation, for it was evident before coagulation took place; nor on the frothy state in which it is usually obtained, (as alleged by Dr. J. Davy,) for in their experiment the frothing was prevented.

To ascertain whether, as is stated by Dr. Davy, carbonic acid is absorbed in greater quantity by the serum of blood than by water, the authors added carbonic acid in successive quantities to the arterial blood employed in the previous experiment without removing the clot. The absorption was gradual, as no agitation was used, the temperature between 5° and 10° Cent. (40° and 50° Fahr.) In five days, one hundred measures of blood had absorbed one hundred and twenty of gas, and in ten weeks more, sixteen additional measures of gas were absorbed, in all, one hundred and thirty-six. In a subsequent experiment, one hundred measures of arterial blood absorbed in three weeks one hundred and forty of carbonic acid, and one hundred of venous blood absorbed in the same time one hundred and eleven of gas.

The next experiment was to determine whether the blood contains carbonic acid in combination with an alkali. Blood from the vein of a dog was collected over mercury as before, but in two separate quantities, both of which were submitted to the action of the air pump, after one of them had been mixed with concentrated vinegar. The same was done with arterial blood. In the jars containing pure blood, the mercury, which was an inch higher than in the former experiment, began to fall when the exhaustion reached twenty-six and a half inches, and, as before, the resulting vacuum disappeared the instant a little air was re-admitted. The arterial blood which had been mixed with vinegar, gave out, at an exhaustion of twenty inches, many small bubbles of air, which at twenty-five inches occupied a space equal to one-third of the volume of the blood employed. On re-admitting the air, a small quantity of gas remained for a few moments, and then almost wholly disappeared. The same took place in the acidulated venous blood, but the gas appeared at twenty-three and a half inches of exhaustion, and at twenty-five inches occupied a space equal to the whole volume of the blood; on re-admission of the air, larger gas bubbles remained than in the arterial blood, though the quantity of venous blood employed was less.

From this experiment it was inferred that carbonic acid, combined with a base, is contained both in arterial and venous blood, but in larger proportion in the latter. For greater certainty, however, another experiment was made, by heating a portion of each kind of blood in a flask with vinegar, and passing the gas evolved through barytic water; from the respective quantities of carbonate of baryta obtained, it appeared that ten thousand parts of venous blood contains at least 12.3, and ten thousand of arterial 8.3 of combined carbonic acid; so that, the proportion in venous blood is to that in arterial as three to two.

The authors next compare the result of their experiments with the leading views entertained as to the formation of carbonic acid emitted during respiration. They observe that the facts are obviously more favorable to the opinion that the carbonic acid is formed in the lungs, by direct combination of its constituents, than to the opposite theory of Lagrange, with which indeed they are scarcely at all reconcileable; but they regard both views as unsatisfactory, and suggest one of their own, which

* Phil. Trans. 1835, p. 2. † Handbuch der Physiologie, p. 315.

they think may form the foundation of a theory of respiration more consistent with known facts.

They first remark that acetic or lactic acid exists in the blood, and in most animal secretions, either free or combined with an alkali; that it is excreted with the sweat and urine in such quantity as cannot be accounted for by supposing it to be introduced with the aliment, and that it must therefore be formed in the body. As, moreover, this acid is produced in most organic fluids, on exposing them to the air, and this process is greatly favoured by a warm temperature, they consider it highly probable that the acetic or lactic acid naturally existing in the body, is produced by the action of oxygen on the blood in the lungs, where the conditions necessary or favorable to the process are present. Proceeding on this view, the authors offer the following theory of the chemical process of respiration. The air in the lungs permeates the coats of the vessels, and comes into immediate contact with the blood. Part of the oxygen consumed unites directly with carbon and hydrogen, producing a portion of carbonic acid and watery vapour; another part combines with some of the organic constituents of the blood. From both causes new products are formed in that fluid, the chief of which is lactic or acetic acid. This acid decomposes a part of the carbonate of soda, and disengages its carbonic acid, which is expelled. The acetate of soda formed in the lungs is freed of its acetic acid by means of the various secretions, especially the urine and sweat; the alcali combines again with carbonic acid, formed by the further decomposition of the organic constituents of the blood, which takes place in its progress through the body, and arrives again at the lungs as carbonate of soda.

This theory is in strict accordance with the fact that venous blood contains more alkaline carbonate than arterial, and that it contains no carbonic acid; but it might perhaps be expected that a portion of the free carbonic acid disengaged should be retained by the arterial blood. In anticipation of this objection, the authors remark that only a part of the blood which passes through the lungs can be acted on by the air, and only this part can carry along with it free carbonic acid, and that when this is mixed with the unchanged blood, its free carbonic acid becomes fixed by uniting with the carbonate of soda, and forming a bicarbonate.

The authors by no means offer this theory as perfect, and they freely admit that it is still attended with many difficulties. We may remark that it seems to accord well with the facts respecting the colour of the blood recently discovered by Dr. Stevens. From his experiments, confirmed by those of Dr. Turner and others, it appears that the crassamentum of blood, whether arterial or venous, when wholly deprived of its serum by ablution with water, is always of a dark colour, and that in this condition it is not reddened by exposure to oxygen, but that it acquires a florid hue on restoring the serum, or immersing the clot in a saline solution, such as that of sea salt or bicarbonate of soda. The florid colour of arterial blood appears, therefore, to be due to the saline matter of the serum. Dr. Stevens ascribes the colour of venous blood to the supposed presence of carbonic acid, (which, however, is at least very doubtful,) which, like other acids, darkens it; and he conceives that the red colour acquired by the blood in its conversion from venous to arterial, is owing merely to the elimination of this gas, and consequent restoration of the influence of the salts of the serum; the oxygen he supposes to possess a peculiar power of extracting the carbonic acid and, further than this, to have no influence in producing the change of colour. Now, admitting that the red colour depends on saline matter, and, without attempting to account for the dark colour of venous blood, we may observe, that if the action of oxygen in respiration be really such as is supposed by Gmelin and Tiedemann, their theory will go some way to explain the effect of that gas on the colour of the blood; for it is supposed to give rise to acetate and bicarbonate of soda, which salts, if present, must heighten the colour.

### 2. *Examination of the Blood after Extirpation of the Kidneys.*

The well known experiment of Prevost and Dumas is of such importance in regard to the theory of secretion, that, notwithstanding the successful repetition of it by Vauquelin and Segalas, the authors deemed it expedient to repeat it once more. They accordingly removed the right kidney of a dog, by an incision in the lumbar region. Neither urine nor excrement was passed for the first twenty-four hours after the operation, but soon after this, both were freely discharged. The wound was completely healed in fourteen days, and the animal appeared, in all respects, as well as before the operation. Twenty-eight days after removal of the right kidney, the left was extirpated. It appeared very vascular, and was about a third larger than the right one, probably from increased activity. The animal survived the second operation only two days. During this time, it occasionally took a little milk and water, twice passed thin greenish yellow excrement, and vomited frequently, first a bilious, and subsequently a watery fluid, with dirty grey mucus. The animal was much dejected, and, shortly before death, was attacked with frequent and violent shiverings.

On opening the body, the peritoneum was found inflamed, and contained a purulent fluid. The vessels of the stomach and intestines were distended with blood, and the mucous membrane of the former appeared inflamed. Both stomach and intestines contained a mucous fluid tinged with bile. The liver was enlarged, very vascular, and very soft and friable, the gall-bladder distended with dark green bile. The right cavities of the heart and venæ cavæ contained dark coagulated blood. The lungs and spleen were natural. The ventricles of the brain contained more fluid than usual.

The following matters were examined chemically with a view to discover urea in them, viz.: 1. The matters vomited. 2. The blood collected from the great vessels amounting to about two ounces. 3. The bile. 4. The contents of the small intestines. 5. The discharged excrement. These substances being dried, were treated with boiling water, and the liquor filtered and precipitated with acetate of lead; the excess of lead being removed in the case of the first three by carbonate of ammonia, and in the last two by sulphuretted hydrogen; the filtered liquor was evaporated to dryness, and treated with alcohol. The residue, obtained by evaporating the alcoholic solution, was then dissolved in a small quantity of water, and into this solution, cooled by means of ice, concentrated nitric acid was slowly dropped, with a result which, in the different substances, was as follows.

From the blood a yellowish white crystalline precipitate, equal in volume to half the mixture: a portion of this precipitate, heated in a platina spoon, left a trace of carbonaceous matter, which disappeared without residue; another portion, gently heated with hydrate of potass, afforded no ammonia. The thick and largest portion was heated with water and carbonate of baryta, and the mixture treated with a large quantity of alcohol. The filtered alcoholic liquor which was not precipitated by sulphuric acid, gave, by spontaneous evaporation, long colourless needles of urea, amounting only to two or three milligramms, but in sufficient quantity to be tried by the usual tests.

The liquor obtained from the vomitings gave a precipitate with nitric acid, resembling in aspect nitrate of urea, but in too small quantity to allow of its nature being determined with certainty. The precipitate obtained from the bile had no resemblance to nitrate of urea. The liquor obtained from the contents of the intestine and the excrement gave no precipitate with nitric acid.

From this it follows that urea was certainly present in the blood, and probably so in the matters vomited; but that it was not detected in the bile, the contents of the intestine, nor the fæces. The small quantity of urea found in the blood, compared with that obtained by Prevost and Dumas, is explained partly by the small quantity of blood analyzed, and partly by the shortness of the time the animal lived after the operation.

3. *Unsuccessful attempt to discover Urea and Sugar of Milk in Healthy Blood.*

It having been first ascertained that 0.2 grammes of urea and 0.5 grammes of sugar of milk, each purposely mixed with fifty grammes of cow's blood, could with certainty be detected by the usual process, it appeared reasonable to expect that, by operating on large quantities of blood, these ingredients, if present, might be discovered, though existing in still smaller proportion. Accordingly, ten pounds of blood from a cow giving milk, were submitted to experiment, but neither urea nor sugar of milk could be discovered in it. From this, it follows that healthy cow's blood contains neither of these substances, or that they are present in it in so small proportion as to be concealed by the other ingredients, and rendered indistinguishable by the usual process.

*Zeitschrift für Physiologie*, B. v. Heft 1.

